# Supplementary material for: Transcriptome Analysis Reveals the Response Mechanism of Digitaria sanguinalis, Arabidopsis thaliana and Poa annua under 4,8-Dihydroxy-1-tetralone Treatment
Source: Plants (Basel). 2023 Jul 22;12(14):2728. doi: 10.3390/plants12142728 (PMC10385526; doi:10.3390/plants12142728)
Supplement: Supplementary file 1 [file plants-12-02728-s001.zip › plants-2382127-supplementary.pdf]

Supplementary Materials

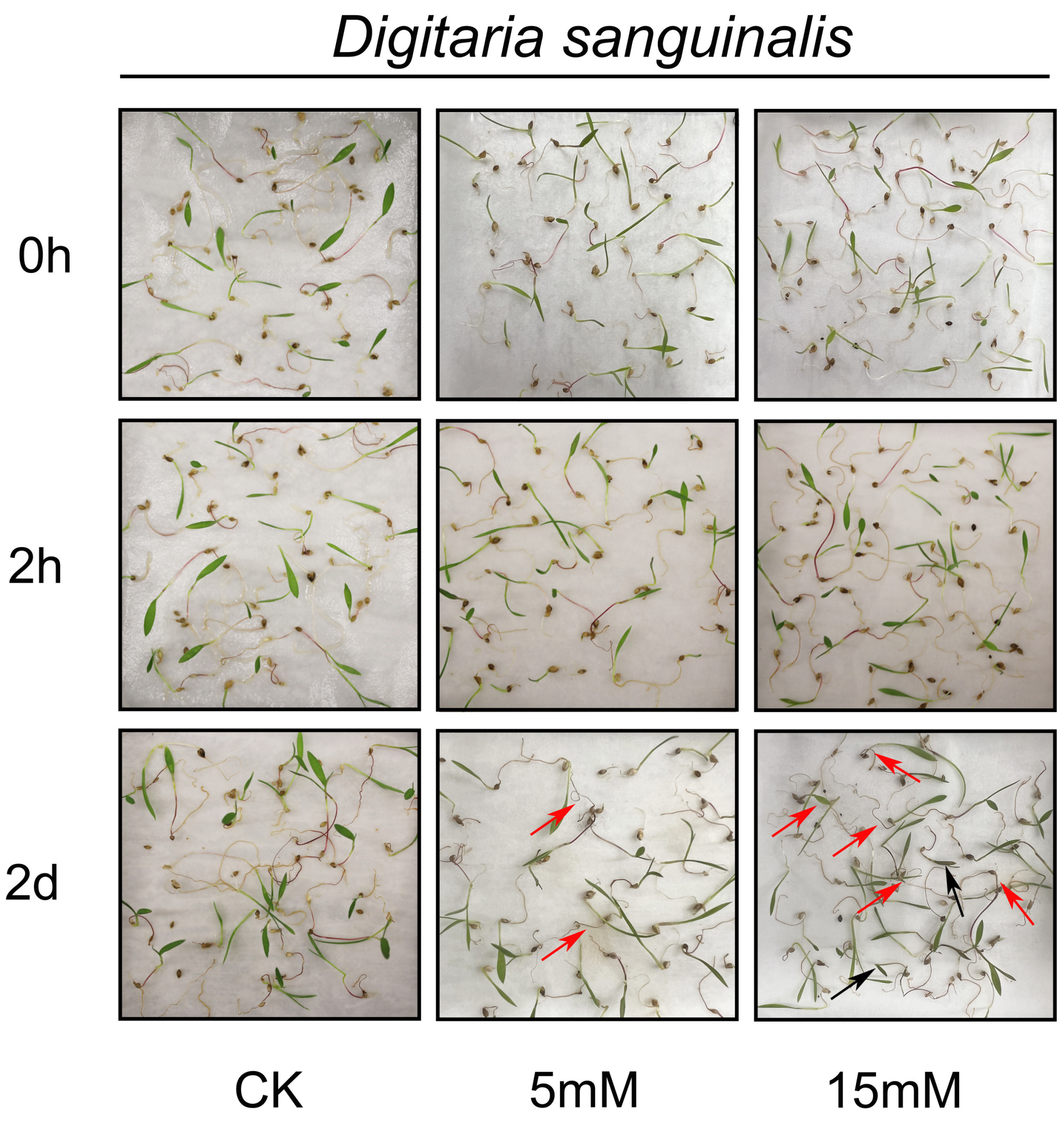

Figure S1. The phenotype of *Digitaria sanguinalis* after the treatment of 4,8-DHT. The 7-day old seedlings of *Digitaria sanguinalis* were treated with 0 mM, 5 mM and 15 mM 4,8-DHT solution for 0 h, 2 h and 2 d, respectively. Red arrows indicate severely browned plant roots and yellowing plant leaves and black arrows indicate dead plants.

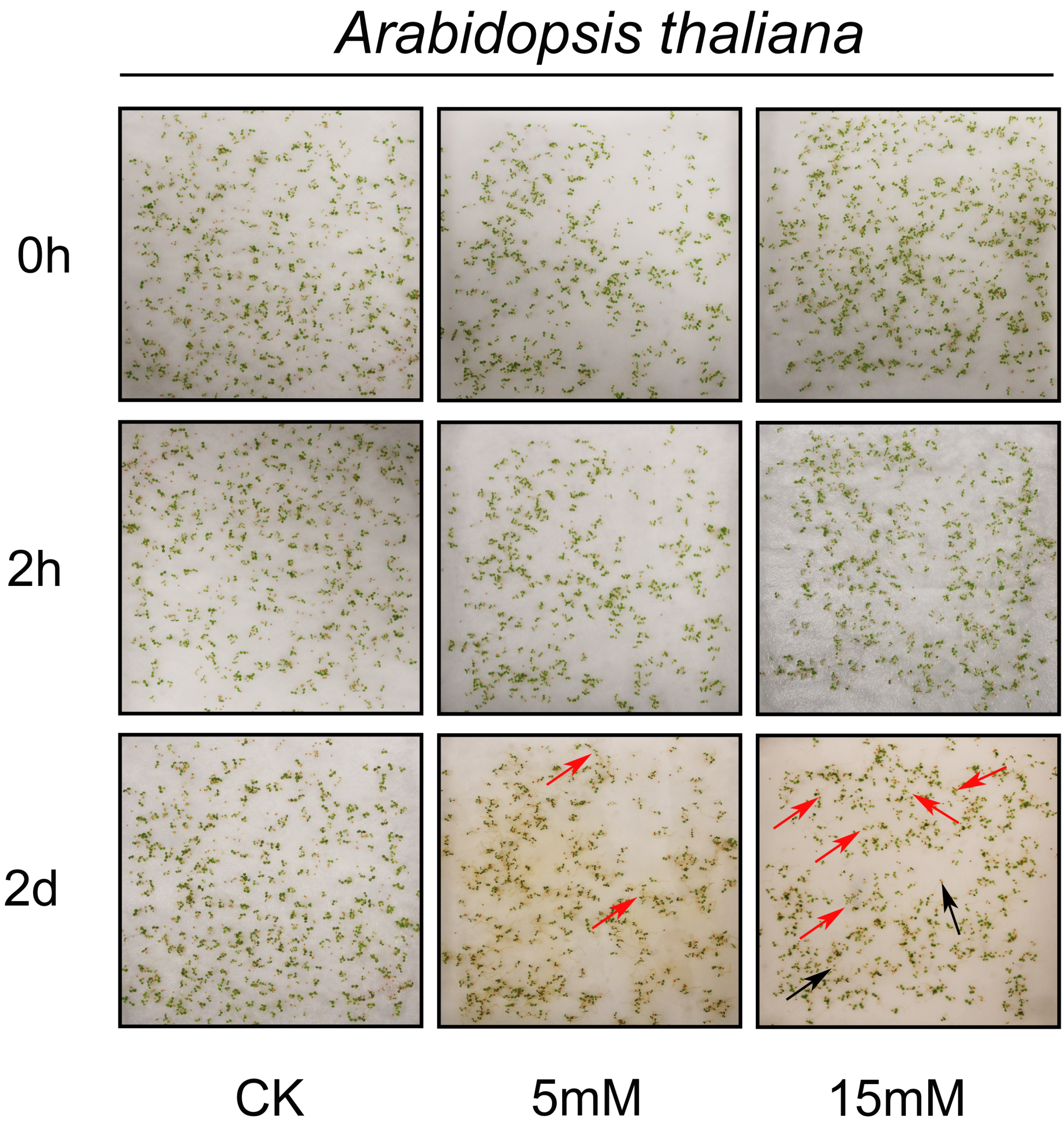

Figure S2. The phenotype of *Arabidopsis thaliana* after the treatment of 4,8-DHT. The 7-day old seedlings of *Arabidopsis thaliana* were treated with 0 mM, 5 mM and 15 mM 4,8-DHT solution for 0 h, 2 h and 2 d, respectively. Red arrows indicate severely browned plant roots and yellowing plant leaves and black arrows indicate dead plants.

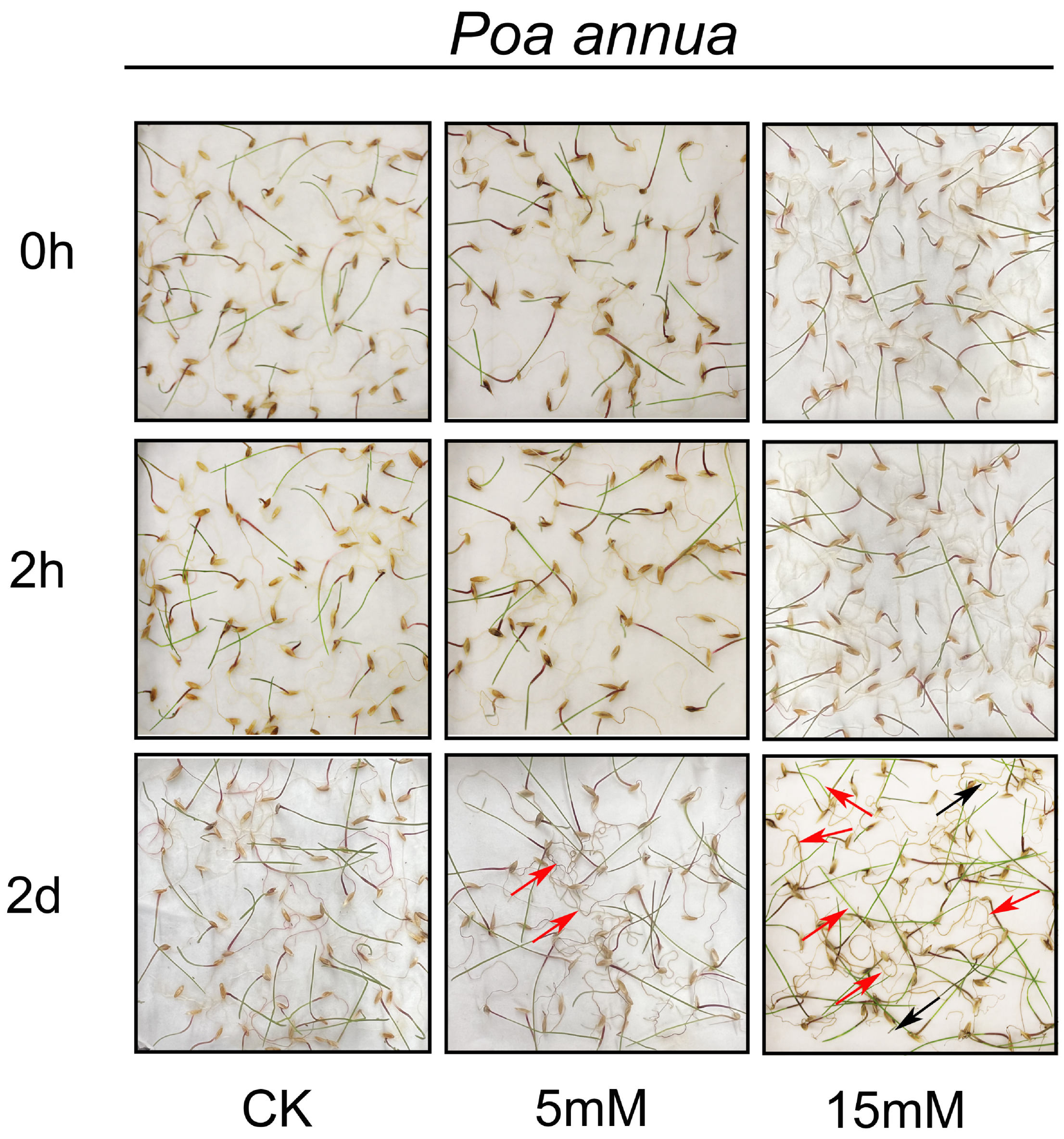

Figure S3. The phenotype of *Poa annua* after the treatment of 4,8-DHT. The 7-day old seedlings of *Poa annua* were treated with 0 mM, 5 mM and 15 mM 4,8-DHT solution for 0 h, 2 h and 2 d, respectively. Red arrows indicate severely browned plant roots and yellowing plant leaves and black arrows indicate dead plants.

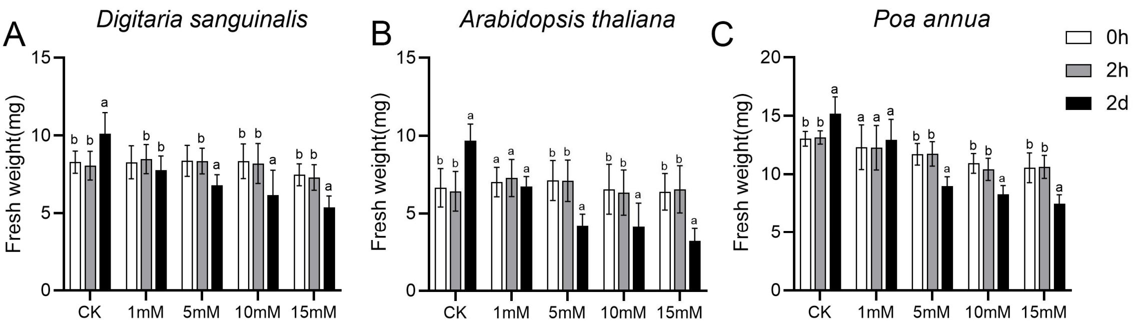

Figure S4. Effect of 4,8-DHT treatment on fresh weight of different species. Measurements of *Digitaria sanguinalis* (**A**), *Arabidopsis thaliana* (**B**) and *Poa annua* (**C**) fresh weights at 0 h, 2 h and 2 d with 0 mM, 5 mM and 15 mM 4,8-DHT treatment, respectively. Bars represent the mean ± SD of at least three independent experiments. Different letters indicate a significant difference among the 4,8-DHT concentration or treatment time ($P < 0.05$) by Tukey's test.

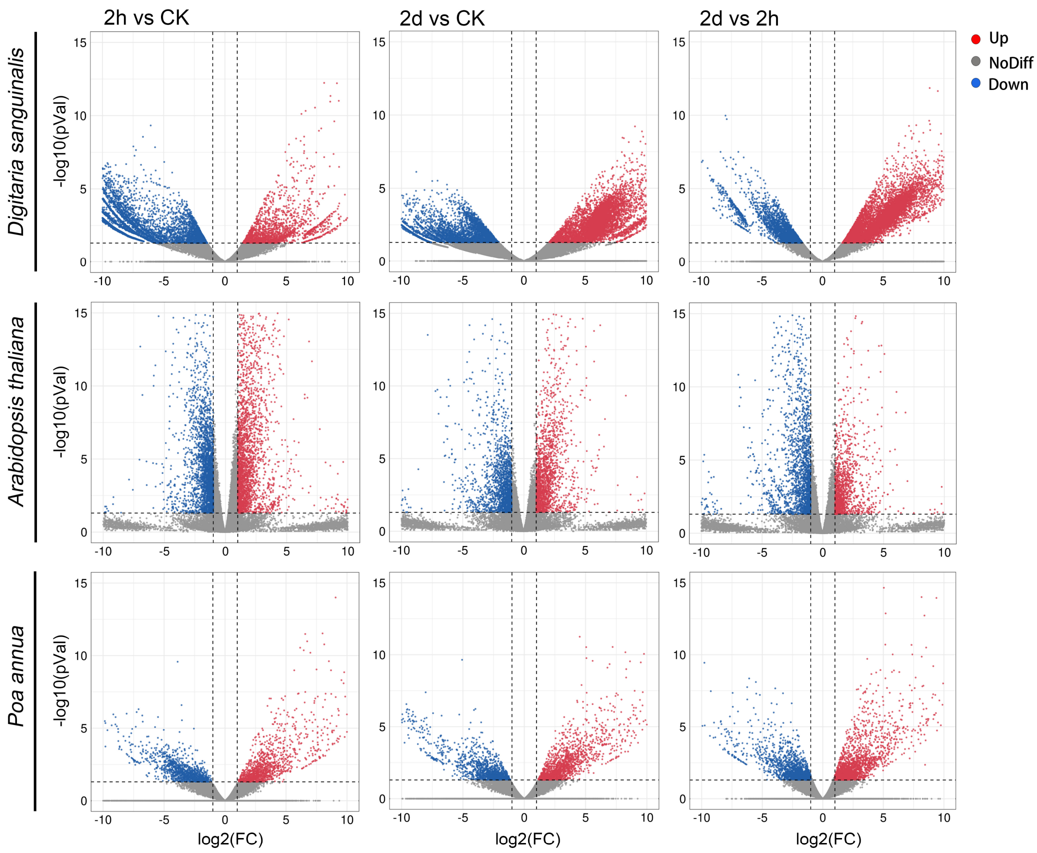

Figure S5. Volcano plots for *Digitaria sanguinalis* (*Ds*), *Arabidopsis thaliana* (*At*) and *Poa annua* (*Pa*) after the treatment of 5 mM 4,8-DHT at 2h vs CK, 2d vs CK and 2d vs 2h, respectively.

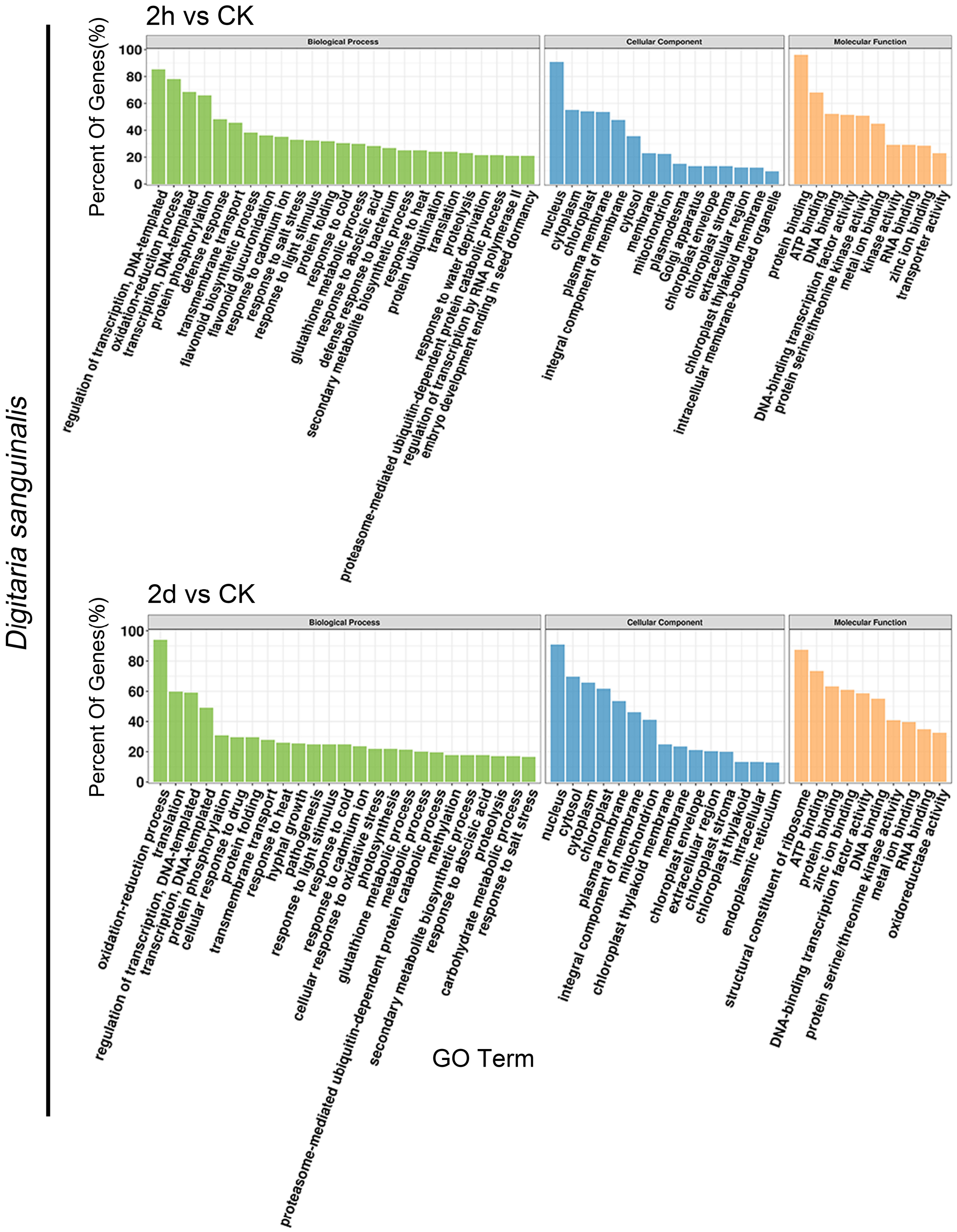

Figure S6. The top 50 GOs for *Digitaria sanguinalis* at the 2h vs CK and 2d vs CK. The x-axis indicates the number of genes per GO term as a percentage of the total number of genes, and the y-axis indicates the enriched GO terms. Different colors are used to distinguish between biological processes, cellular components and molecular functions.

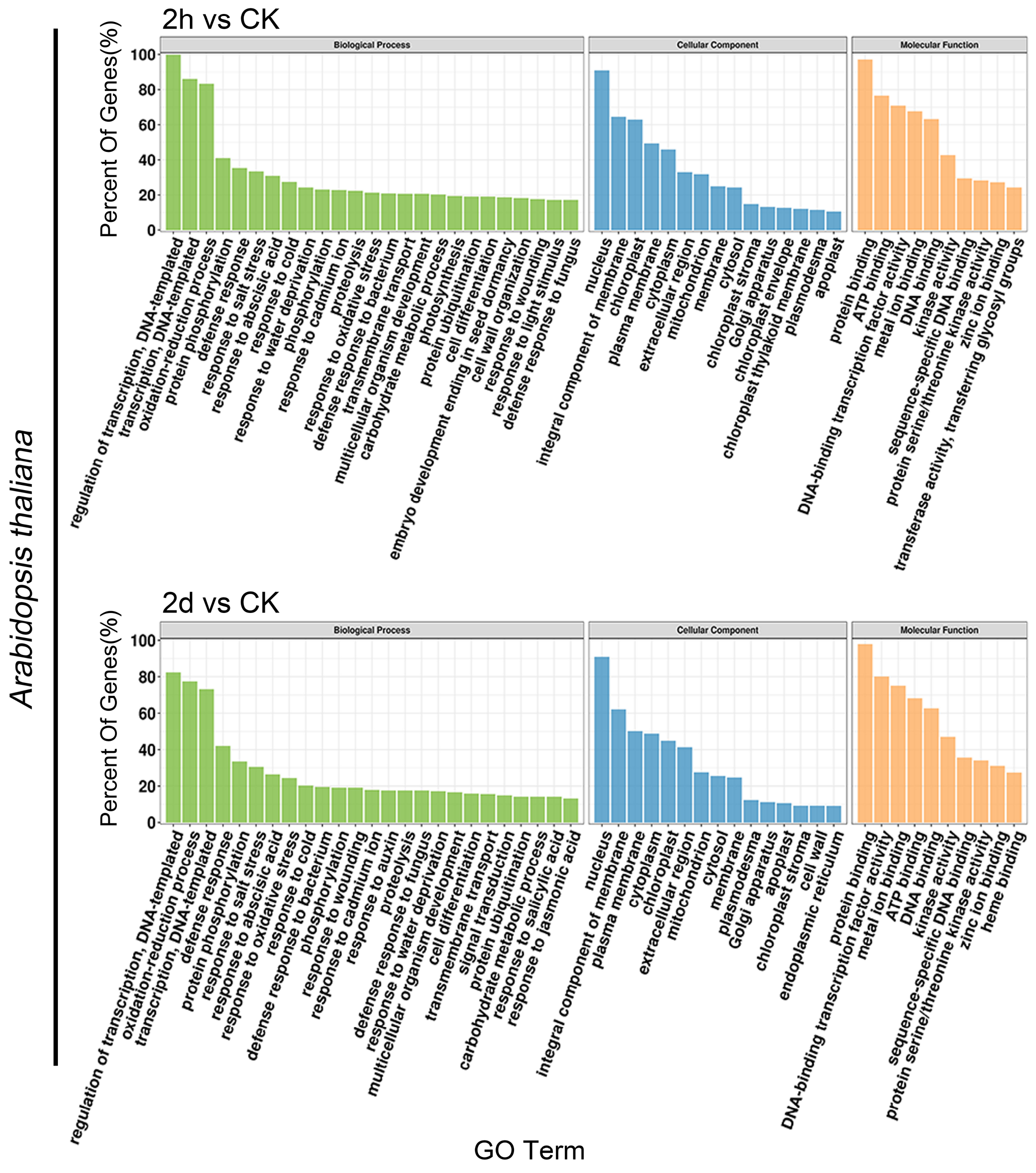

Figure S7. The top 50 GOs for *Arabidopsis thaliana* at the 2h vs CK and 2d vs CK. The x-axis indicates the number of genes per GO term as a percentage of the total number of genes, and the y-axis indicates the enriched GO terms. Different colors are used to distinguish between biological processes, cellular components and molecular functions.

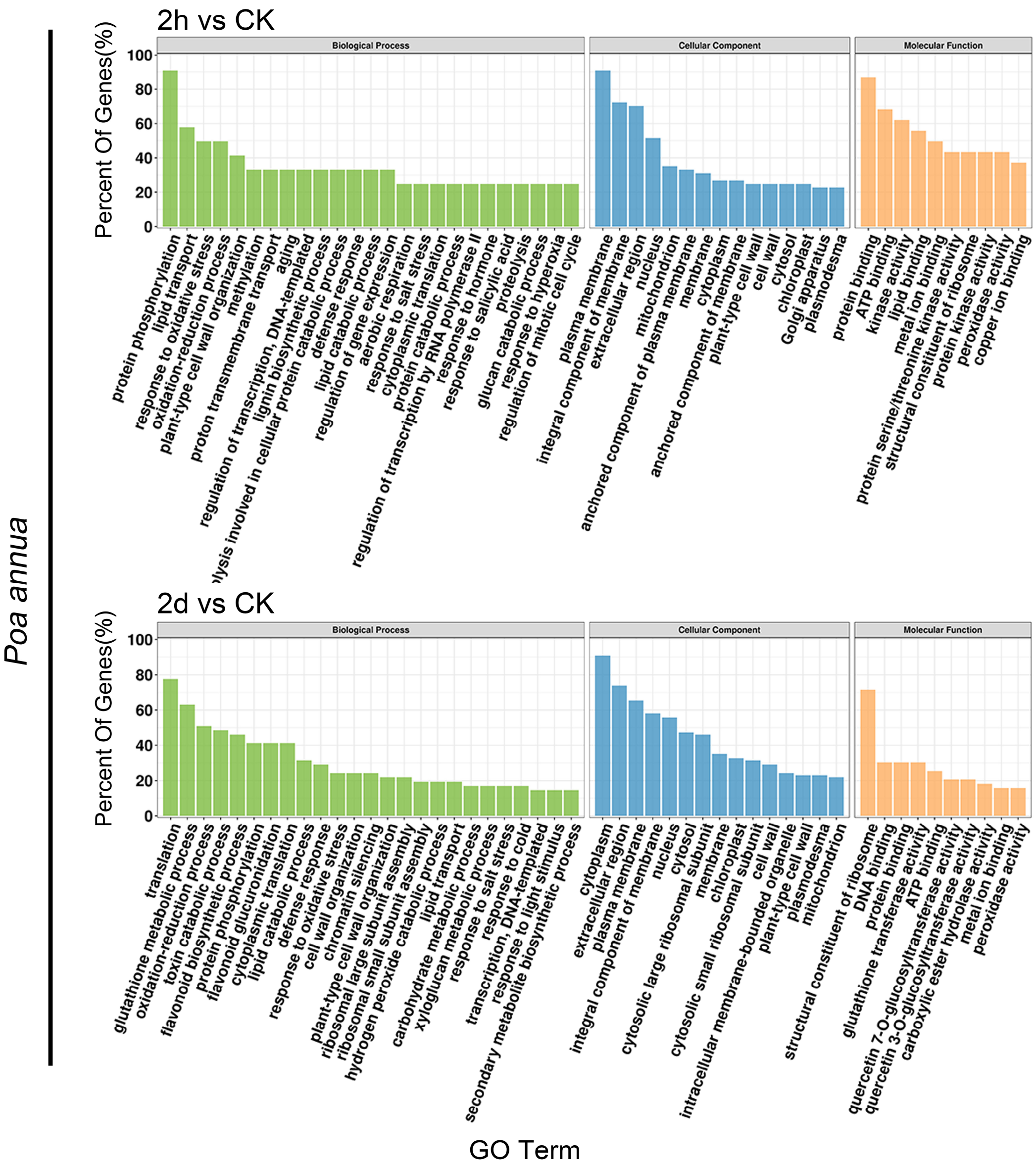

Figure S8. The top 50 GOs for *Poa annua* at the 2h vs CK and 2d vs CK. The x-axis indicates the number of genes per GO term as a percentage of the total number of genes, and the y-axis indicates the enriched GO terms. Different colors are used to distinguish between biological processes, cellular components and molecular functions.

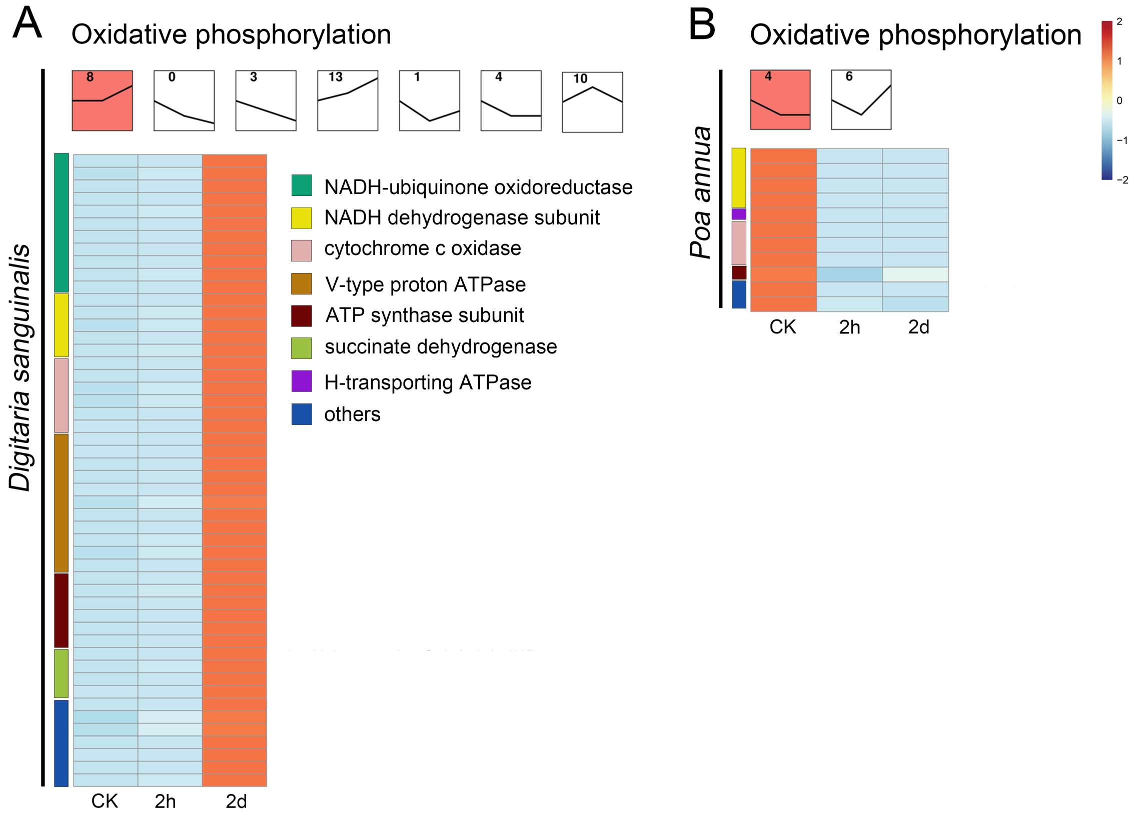

Figure S9. STEM plots and heatmaps of DEGs in oxidative phosphorylation of *Digitaria sanguinalis* (**A**) and *Arabidopsis thaliana* (**B**) after the 0h, 2h and 2d treatment of 5 mM 4,8-DHT. STEM has multiple temporal expression patterns, and the colored modules represent the most significant among these DEGs. The different-colored squares on the left of the heatmaps correspond to the genes of the same-colored squares on the right. The different number in the square only indicates the serial number of the expression patterns.

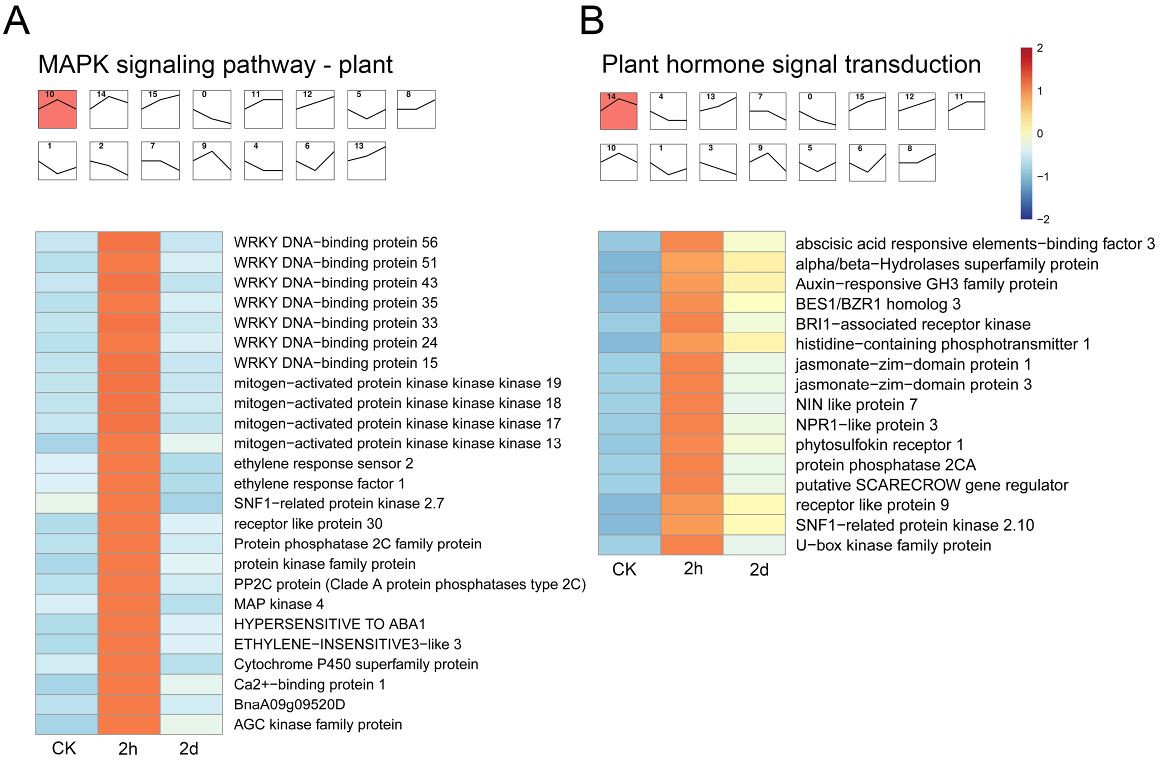

Figure S10. Changes in the DEGs associated with the resistance to oxidative stress in *Arabidopsis thaliana* (*At*) after the 0h, 2h and 2d treatment of 4,8-DHT. (**A**). STEM plots and heatmaps of DEGs are involved in the plant hormone signal transduction; (**B**). STEM plots and heatmaps of DEGs are involved in the MAPK signaling pathway. STEM has multiple temporal expression patterns, and the colored modules represent the most significant among these DEGs. The different number in the square only indicates the serial number of the expression patterns.

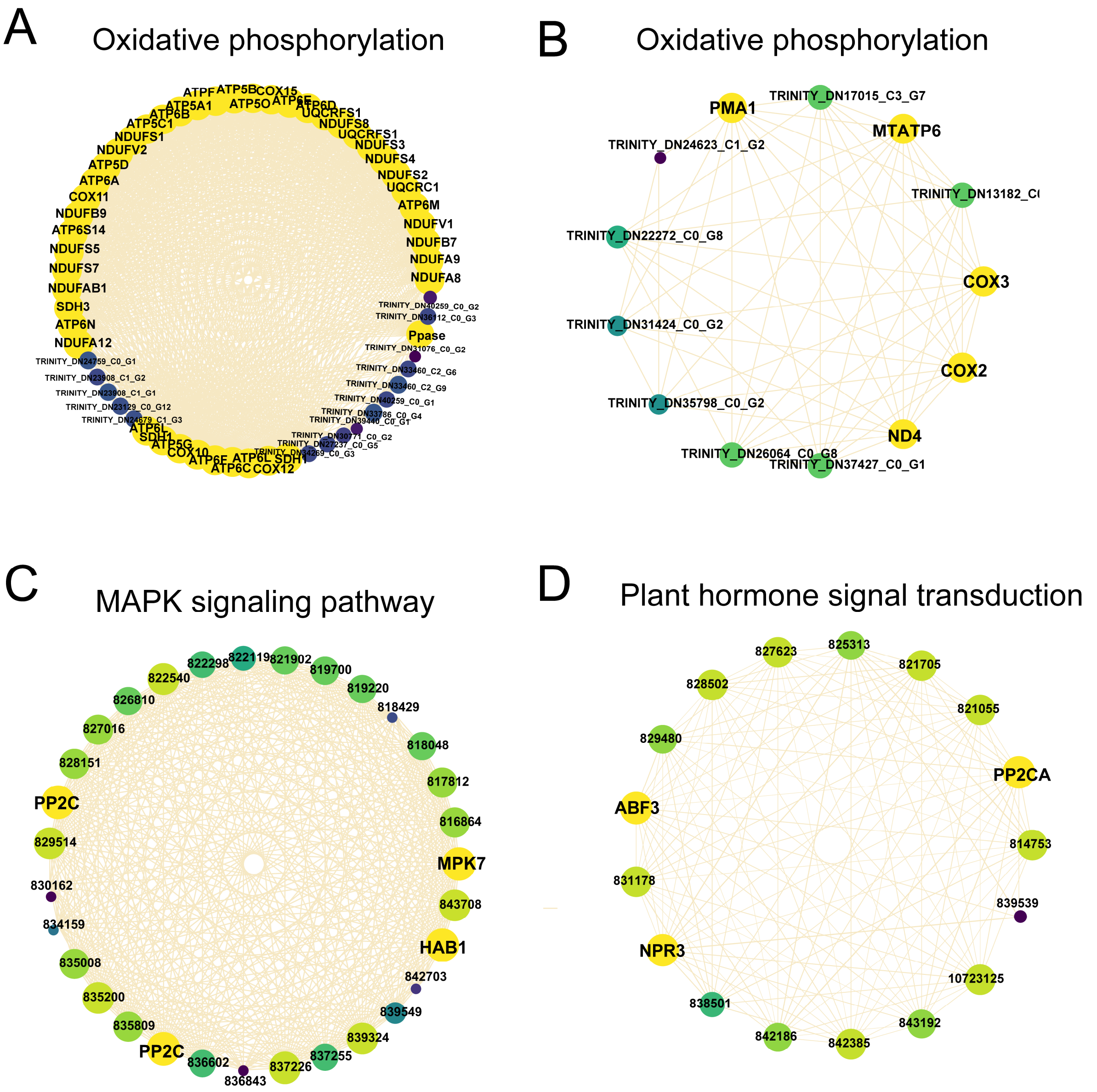

Figure S11. Correlation networks for oxidative phosphorylation, MAPK signaling pathway and plant hormone signal transduction in *Digitaria sanguinalis (Ds)*, *Arabidopsis thaliana (At)* and *Poa annua (Pa)*. (**A**). Correlation networks for *Ds* are involved in oxidative phosphorylation; (**B**). Correlation networks for *Pa* are involved in oxidative phosphorylation; (**C**). Correlation networks for *At* are involved in MAPK signaling pathway; (**D**). Correlation networks for *At* are involved in plant hormone signal transduction. Yellow represents key genes, and genes with higher linkage counts in the corresponding networks are shown in the larger circles.

Table S1. Key genetic information for correlation network analysis of photosynthetic pathways in *Digitaria sanguinalis (Ds)* and *Arabidopsis thaliana (At)*.

Species	Gene ID	Gene Name	Gene Annotation
	Photosynthesis		
Digitaria sanguinalis	TRINITY_DN34418_c0_g1	*PetC*	cytochrome b6-f complex iron-sulfur subunit, chloroplastic isoform X2
	TRINITY_DN27033_c1_g2	*ATPF1G*	ATP synthase subunit gamma, chloroplastic
	TRINITY_DN27438_c0_g1	*PsaH*	photosystem I reaction center subunit VI
	TRINITY_DN35842_c0_g1	*Psb28*	photosystem II reaction center PSB28 protein,
	TRINITY_DN30137_c1_g1	*PsbY*	photosystem II core complex proteins psbY
	TRINITY_DN33614_c0_g2	*ATPG*	ATP synthase subunit b', chloroplastic
	TRINITY_DN30034_c0_g2	*PsaG*	photosystem I reaction center subunit V, chloroplastic
	TRINITY_DN33317_c0_g5	*PsaD*	photosystem I subunit II
Arabidopsis thaliana	818532	*PPL2*	PsbP-like protein 2
	821143	*PsbQ*	PsbQ-like 1
	824699	*PPL1*	PsbP-like protein 1
	825797	*ATPC1*	ATPase, F1 complex, gamma subunit protein
	825866	*PsbQ*	photosystem II subunit Q-2
	827877	*PsbQ*	photosystem II subunit QA
	827996	*PetC*	photosynthetic electron transfer C
	828984	*Psb28*	photosystem II reaction center PSB28 protein
	829359	*ATPF0B*	ATPase, F0 complex, subunit B/B', bacterial/chloroplast
	836751	*FNR1*	ferredoxin-NADP^{+} reductase
	837974	*PsbQ*	PsbQ-like 2
	838591	*FNR2*	ferredoxin-NADP [+]-oxidoreductase 2
	838622	*PetE*	Cupredoxin superfamily protein
	839449	*Psb27*	photosystem II family protein
	828183	*PsaD*	photosystem I subunit D-1
	834534	Trypsin family protein	Trypsin family protein
	838876	TPR-like superfamily protein	Tetratricopeptide repeat (TPR)-like superfamily protein
	Photosynthesis - antenna proteins		
Digitaria sanguinalis	TRINITY_DN32282_c0_g1	*LHCA1*	chlorophyll a-b binding protein 1B-21, chloroplastic
	TRINITY_DN35117_c0_g1	*LHCB4*	chlorophyll a-b binding protein CP29.1, chloroplastic
	TRINITY_DN28327_c0_g2	*LHCA4*	chlorophyll a-b binding protein P4, chloroplastic
	TRINITY_DN38985_c0_g1	*LHCB7*	chlorophyll a-b binding protein 7, chloroplastic
Arabidopsis thaliana	820043	*LHCB4.2*	light harvesting complex photosystem II
	835515	*LHCB3*	chlorophyll a-b binding protein 3, chloroplastic
	838151	*LHCB6*	light harvesting complex photosystem II subunit 6

	839869	*LHCB3*	chlorophyll A/B binding protein 3
	Carbon fixation		
Digitaria sanguinalis	TRINITY_DN38874_C1_G1	*Rubisco*	ribulose bisphosphate carboxylase small chain A
	TRINITY_DN25725_C2_G1	*GAPA*	glyceraldehyde-3-phsphate dehydrogenase A
	TRINITY_DN22992_C0_G1	*PEPC*	C4 phosphoenolpyruvate carboxylase
Arabidopsis thaliana	833829	*Rubisco*	Ribulose bisphosphate carboxylase (small chain) family protein
	843565	*ALT2*	alanine aminotransferase 2

Table S2. Key genetic information for correlation network analysis of phenylpropanoid biosynthesis and glutathione metabolism in *Digitaria sanguinalis (Ds)*, *Arabidopsis thaliana (At)* and *Poa annua (Pa).*

Species	Gene ID	Gene Name	Gene Annotation
	Phenylpropanoid biosynthesis		
Digitaria sanguinalis	TRINITY_DN27015_c0_g3	*POD A2*	peroxidase A2
Arabidopsis thaliana	829907	*POD*	Peroxidase superfamily protein
Poa annua	TRINITY_DN21631_c0_g4	*POD43*	Peroxidase 43
	Glutathione metabolism		
Digitaria sanguinalis	TRINITY_DN31220_c1_g1	*GSTT1*	Glutathione S-transferase T1
	TRINITY_DN28189_c0_g2	*NDHL*	NAD(P)H-quinone oxidoreductase subunit L
	TRINITY_DN28158_c2_g6	*GSTU6*	glutathione S-transferase 1
	TRINITY_DN27465_c0_g4	*GPX*	putative phospholipid hydroperoxide glutathione peroxidase 6, mitochondrial
Arabidopsis thaliana	822003	*GSR*	glutathione-disulfide reductase
	826765	*GPX6*	glutathione peroxidase 6
Poa annua	TRINITY_DN35776_c0_g2	*GSTU6*	probable glutathione S-transferase GSTU6
	TRINITY_DN34256_c0_g2	*GSTU6*	Probable glutathione S-transferase GSTU6
	TRINITY_DN30998_c0_g1	*GSTU6*	probable glutathione S-transferase GSTU6
	TRINITY_DN29602_c0_g1	*GSTU6*	putative glutathione S-transferase GSTU6
	TRINITY_DN19080_c1_g3	*GSTU6*	glutathione s-transferase U2

Table S3. Key genetic information for correlation network analysis of oxidative phosphorylation, MAPK signaling pathway and plant hormone signal transduction in *Digitaria sanguinalis (Ds)*, *Arabidopsis thaliana (At)* and *Poa annua (Pa)*.

Species	Gene ID	Gene Name	Gene Annotation
	Oxidative phosphorylation		
Digitaria sanguinalis	TRINITY_DN33695_C0_G1	*NDUFA9*	NADH-ubiquinone oxidoreductase 40 kDa subunit
	TRINITY_DN34085_C0_G1	*Ppase*	inorganic pyrophosphatase-like
	TRINITY_DN25548_C0_G2	*NDUFA8*	NADH-ubiquinone oxidoreductase 20.8 kDa subunit-like
	TRINITY_DN20554_C0_G1	*ATP6M*	V-type proton ATPase subunit D-like
	TRINITY_DN22979_C0_G1	*NDUFV1*	NADH-ubiquinone oxidoreductase 51 kDa subunit, mitochondrial-like
	TRINITY_DN32629_C0_G3	*NDUFB7*	NADH-ubiquinone oxidoreductase B18 subunit
	TRINITY_DN27155_C0_G4	*ATP6L*	V-type proton ATPase 16 kDa proteolipid subunit 2-like
	TRINITY_DN37634_C0_G3	*NDUFS2*	NADH-ubiquinone oxidoreductase 49 kDa subunit, mitochondrial-like
	TRINITY_DN20671_C0_G1	*NDUFV2*	NADH-ubiquinone oxidoreductase 24 kDa subunit, mitochondrial-like
	TRINITY_DN32506_C0_G3	*ATP5D*	ATP synthase subunit delta, mitochondrial-like
	TRINITY_DN25108_C0_G1	*ATP6A*	V-type proton ATPase catalytic subunit A-like
	TRINITY_DN21733_C0_G1	*COX11*	cytochrome c oxidase assembly protein COX11, mitochondrial-like
	TRINITY_DN33762_C0_G1	*COX10*	protoheme IX farnesyltransferase, mitochondrial-like
	TRINITY_DN39201_C1_G1	*SDH1*	probable succinate dehydrogenase [ubiquinone] flavoprotein subunit, mitochondrial
	TRINITY_DN27244_C0_G2	*ATP6S14*	V-type proton ATPase subunit F-like
	TRINITY_DN34269_C0_G3	*UQCRC1*	cytochrome c1, heme protein, mitochondrial-like
	TRINITY_DN36196_C0_G1	*ATP6BATP6B*	V-type proton ATPase subunit B
	TRINITY_DN24551_C0_G2	*ATP5C1*	ATP synthase subunit gamma, mitochondrial-like
	TRINITY_DN23012_C0_G2	*ATP6F*	probable V-type proton ATPase 20 kDa proteolipid subunit
	TRINITY_DN20747_C0_G1	*NDUFA2*	PREDICTED: NADH dehydrogenase [ubiquinone] 1 alpha subcomplex subunit 2
	TRINITY_DN30596_C0_G1	*NDUFS8*	NADH-ubiquinone oxidoreductase 23 kDa subunit, mitochondrial-like
	TRINITY_DN31842_C0_G1	*COX15*	cytochrome c oxidase assembly protein COX15-like
	TRINITY_DN40545_C0_G1	*ATP5A1*	ATP synthase subunit alpha, mitochondrial
	TRINITY_DN36569_C0_G2	*NDUFS1*	NADH-ubiquinone oxidoreductase 78 kDa subunit, mitochondrial-like
	TRINITY_DN25470_C0_G2	*NDUFAB1*	vacuolar protein sorting-associated protein 74-like
	TRINITY_DN22757_C0_G1	*NDUFS7*	NADH-ubiquinone oxidoreductase 19.3 kDa subunit
	TRINITY_DN30729_C1_G4	*ATP6L*	V-type proton ATPase 16 kDa proteolipid subunit [Quercus suber]
	TRINITY_DN22557_C0_G1	*NDUFS5*	NADH dehydrogenase [ubiquinone] iron-sulfur protein 5-B-like
	TRINITY_DN22393_C0_G2	*COX12*	cytochrome c oxidase subunit 6B-like
	TRINITY_DN22308_C0_G1	*NDUFB9*	NADH dehydrogenase (ubiquinone) 1 beta subcomplex subunit 9
	TRINITY_DN27152_C0_G1	*NDUFA12*	NADH: ubiquinone oxidoreductase 18 kDa subunit
	TRINITY_DN29967_C1_G2	*ATP6C*	V-type proton ATPase subunit C 1-like
	TRINITY_DN33127_C0_G1	*UQCRFS1*	ubiquinol-cytochrome c reductase iron-sulfur subunit
	TRINITY_DN32704_C0_G1	*ATP5O*	ATP synthase subunit 5, mitochondrial-like
	TRINITY_DN39508_C0_G1	*ATP5B*	ATP synthase subunit beta, mitochondrial-like

Poa annua	TRINITY_DN24644_C0_G1	*SDH3*	succinate dehydrogenase cytochrome B subunit, mitochondrial-like
	TRINITY_DN30954_C0_G3	*ATP6N*	V-type proton ATPase subunit a-like
	TRINITY_DN27269_C0_G7	*ATP5G*	ATP synthase subunit 9, mitochondrial-like
	TRINITY_DN40563_C1_G1	*SDH1*	probable succinate dehydrogenase [ubiquinone] flavoprotein subunit, mitochondrial
	TRINITY_DN25823_C0_G1	*ATP6E*	V-type proton ATPase subunit E-like
	TRINITY_DN23908_C1_G3	*ATPF*	ATP synthase protein YMF19
	TRINITY_DN27129_C0_G3	*NDUFS3*	NADH-ubiquinone oxidoreductase 30.4 kDa subunit, mitochondrial-like
	TRINITY_DN26189_C1_G5	*ATP6D*	V-type proton ATPase subunit d-like
	TRINITY_DN35926_C0_G3	*NDUFS4*	NADH-ubiquinone oxidoreductase 21 kDa subunit, mitochondrial-like
	TRINITY_DN10028_C0_G4	*MTATP6*	H-transporting ATPase (mitochondrion)
	TRINITY_DN9456_C0_G1	*COX2*	cytochrome c oxidase subunit 2 (mitochondrion)
	TRINITY_DN10028_C0_G1	*COX3*	cytochrome c oxidase subunit 3 (mitochondrion)
	TRINITY_DN15424_C0_G3	*PMA1*	Plasma membrane ATPase
	TRINITY_DN7227_C0_G1	*ND4*	NADH dehydrogenase subunit 4 (mitochondrion)
	MAPK signaling pathway		
Arabidopsis thaliana	816330	*MPK7*	mitogen-activated protein kinase 7
	828714	*PP2C*	Protein phosphatase 2C family protein
	836040	*PP2C*	PP2C protein (Clade A protein phosphatases type 2C)
	843609	*HAB1*	hypersensitive to ABA1
	Plant hormone signal transduction		
Arabidopsis thaliana	829547	*ABF3*	abscisic acid responsive elements-binding factor 3
	834545	*NPR3*	NPR1-like protein 3
	820314	*PP2CA*	protein phosphatase 2CA
